# Supplementary material for: Gadd45g insufficiency drives the pathogenesis of myeloproliferative neoplasms
Source: Nat Commun. 2024 Apr 6;15:2989. doi: 10.1038/s41467-024-47297-2 (PMC10998908; doi:10.1038/s41467-024-47297-2)
Supplement: Supplementary file 1 — Supplementary Information [file 41467_2024_47297_MOESM1_ESM.pdf]

Supplementary Information

Supplementary Figure 1-12 and Figure Legends

Supplementary Table 1-6

Supplementary Figures and Figure Legends

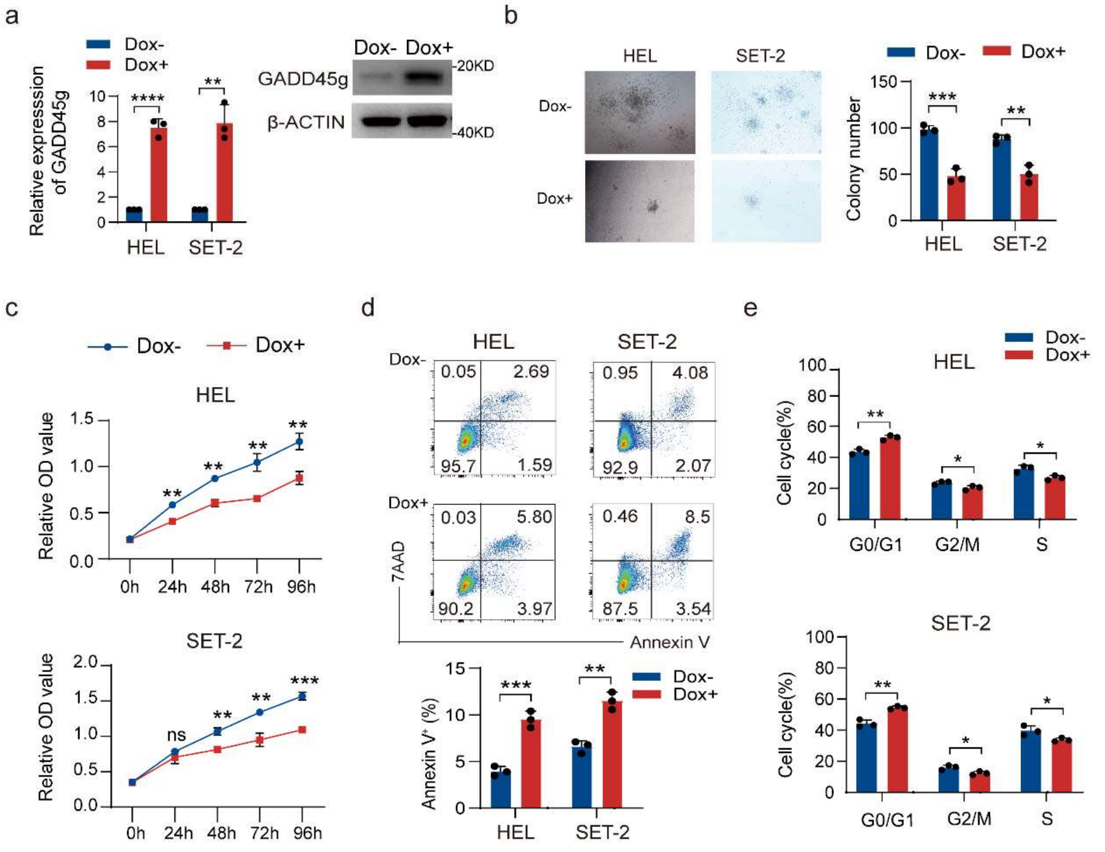

Supplementary Fig. 1 Overexpression of *GADD45g* exhibits anti-tumor activities in human MPNs cells.

(**a**) qRT-PCR evaluation of GADD45g expression in HEL and SET-2 cells with or without Dox-induced *GADD45g* overexpression, and representative Western blot in HEL cells of 3 independent experiments.

(**b-e**) Effects of Dox-induced *GADD45g* expression on colony formation (**b**), cell proliferation (**c**), apoptosis (**d**) and cell cycle (**e**) of HEL and SET-2 cells.

For **a-e**: Figures shown are representative of three independent experiments

with similar results. Data are shown as mean ± SD (n=3 technical replicates). Comparisons were evaluated by two-tailed Student's *t* test, and multiple groups were analyzed with one-way ANOVA. *, $P < 0.05$; **, $P < 0.01$; ***, $P < 0.001$; ns, not significant.

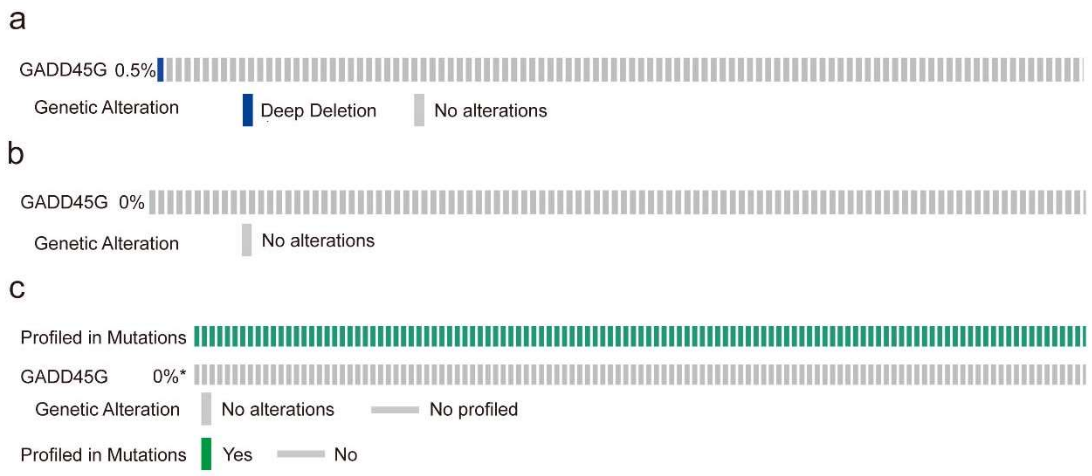

Supplementary Fig. 2 Alteration frequencies of *GADD45g* gene in human myeloid malignancies.

(**a-c**) The genetic alterations of *GADD45g* were analyzed by cBioPortal in a cohort of AML patients (TCGA, PanCancer Atlas, *n* = 190) (http://www.cbioportal.org/) (**a**), a cohort of MPNs patients (CIMR, NEJM 2013, *n* = 151) (**b**), and a cohort of MDSs patients (MSKCC, 2020, *n* = 4231) (**c**).

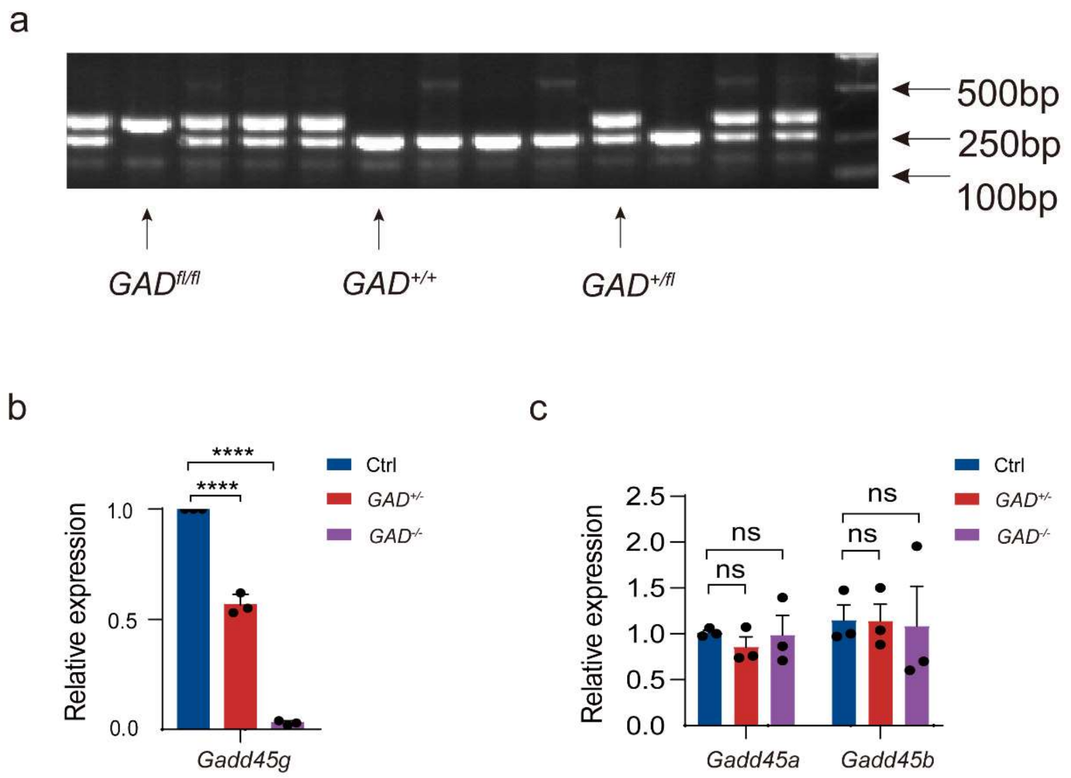

Supplementary Fig. 3 *Gadd45g* deletions in the murine hematopoietic system.

(**a**) Representative gel showing heterozygous and homozygous deletions of *Gadd45g* (*GAD*) in the murine bone marrow mononuclear cells (BMMNCs), as detected by PCR.

(**b**) Relative expression of *Gadd45g* mRNA in BM-derived Lin$^-$c-kit$^+$ cells from *Gadd45g*$^{+/-}$, *Gadd45g*$^{-/-}$ and Ctrl mice, as measured by qRT-PCR (n = 3 mice per group).

(**c**) Relative expression of *Gadd45a* and *Gadd45b* mRNAs in BM-derived Lin$^-$c-kit$^+$ cells from *Gadd45g*$^{+/-}$, *Gadd45g*$^{-/-}$ and Ctrl mice, as determined by qRT-PCR (n = 3 mice per group).

For **b-c**: Data are shown as means ± SD. ****, $P < 0.0001$; ns, not significant (Two-tailed Student's *t* test).

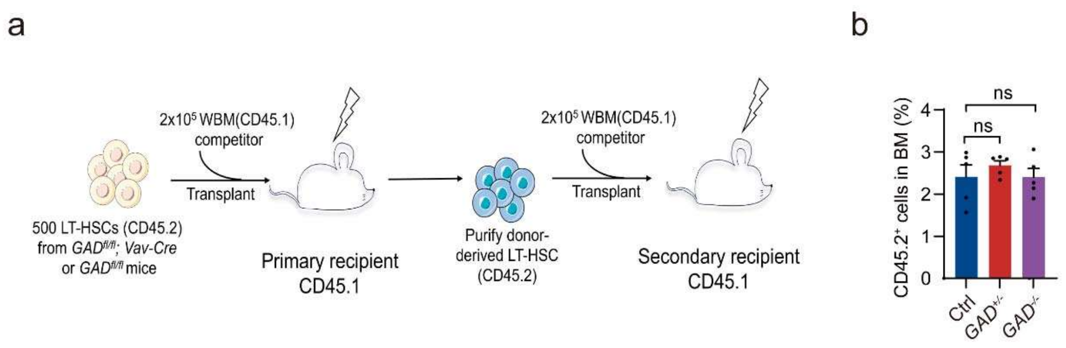

Supplementary Fig. 4 Schema depiction of the bone marrow transplantation and the results of homing tests.

(**a**) Schematic representation of the methods used for bone marrow (BM) transplantation of LT-HSC.

(**b**) Homing assay was performed by intravenous injection of two thousand freshly sorted LT-HSCs from 4-month-old *Gadd45g*$^{+/-}$ (n=5) and *Gadd45g*$^{-/-}$ (n=5) mice or control mice (n=4) into lethally irradiated recipients. Eighteen hours after transplant, bone marrow mononuclear cells (BMMNCs) were collected and CD45.2^{+} cells homing to recipient BM were analyzed by flow cytometry (n=4-5 mice per group).

Data are shown as means ± SD. ns, not significant (Two-tailed Student's *t* test).

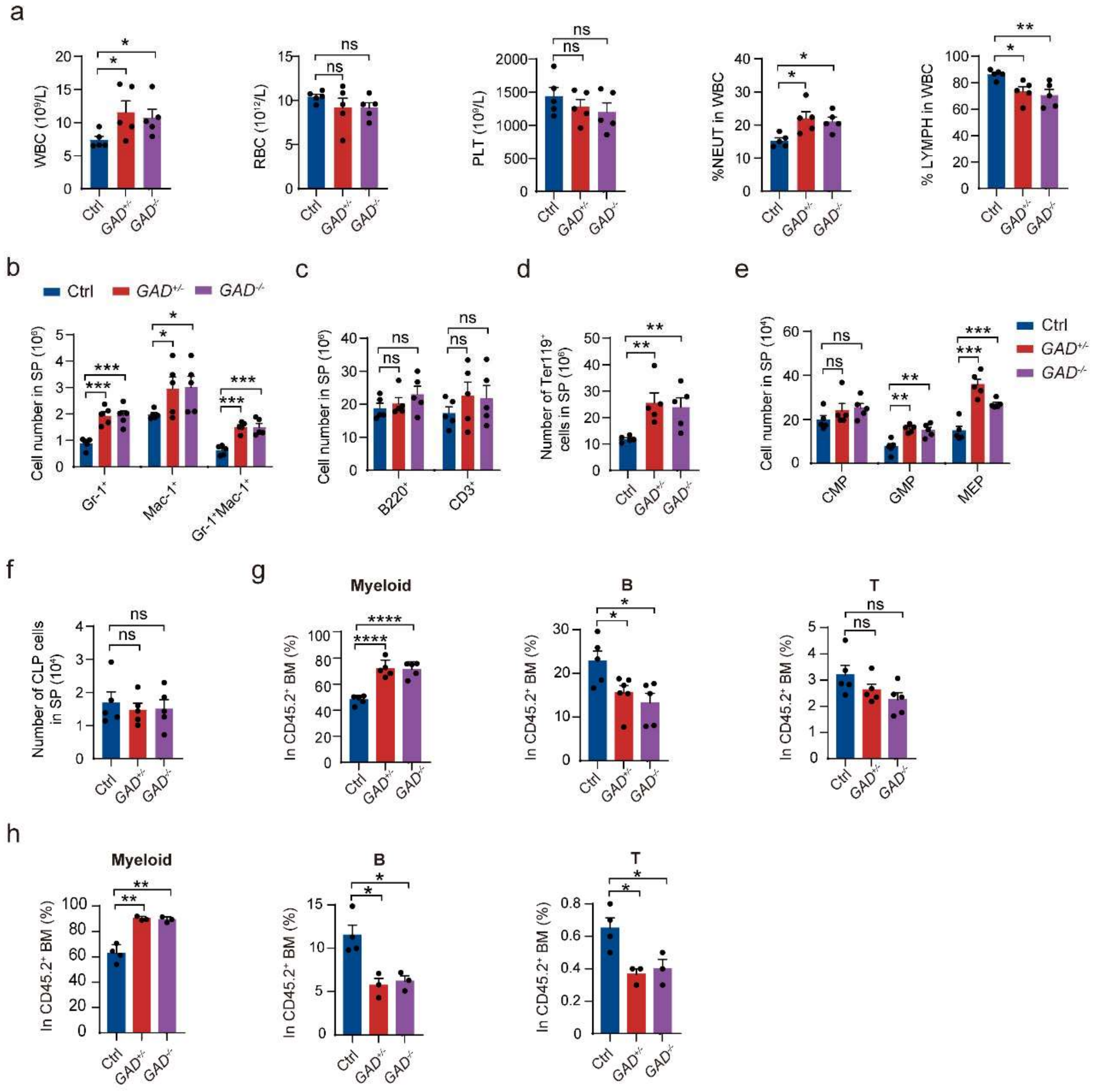

Supplementary Fig. 5 Aberrations of hematological parameters of the PB and spleen from 6-month-old *Gadd45g*-deficient mice and myeloid-biased differentiation in the BM after transplantation.

(**a**) Counts of white blood cell (WBC), red blood cell (RBC) and platelet, and percentages of neutrophils and lymphocytes in the PB of $Gadd45g^{+/-}$, $Gadd45g^{-/-}$ and Ctrl mice (n=5 mice per group).

(**b-f**) Absolute numbers of Gr-1$^+$, Mac-1$^+$ and Gr-1$^+$Mac-1$^+$ cells (**b**), B220$^+$ and

$CD3^+$ cells (**c**), $Ter119^+$ cells (**d**), CMP, GMP, MEP cells (**e**), and CLP cells (**f**) in the spleen of *Gadd45g*$^{+/-}$, *Gadd45g*$^{-/-}$ and Ctrl mice (n=5 mice per group).

(**g-h**) My-biased HSCs from 6-month-old *Gadd45g*$^{+/-}$, *Gadd45g*$^{-/-}$ and Ctrl mice were transplanted together with competitor cells into lethally irradiated recipients. Percentages of donor-derived myeloid (Mac-1^+), B ($B220^+$), and T ($CD3^+$) cells in $CD45.2^+$ BM at 16 weeks after primary BM transplantation (BMT) (**g**) and secondary BMT (**h**) (n=4 mice per group).

Data are shown as means ± SD. *, $P < 0.05$; **, $P < 0.01$; ***, $P < 0.001$; ****, $P < 0.0001$; ns, not significant (Two-tailed Student's *t* test).

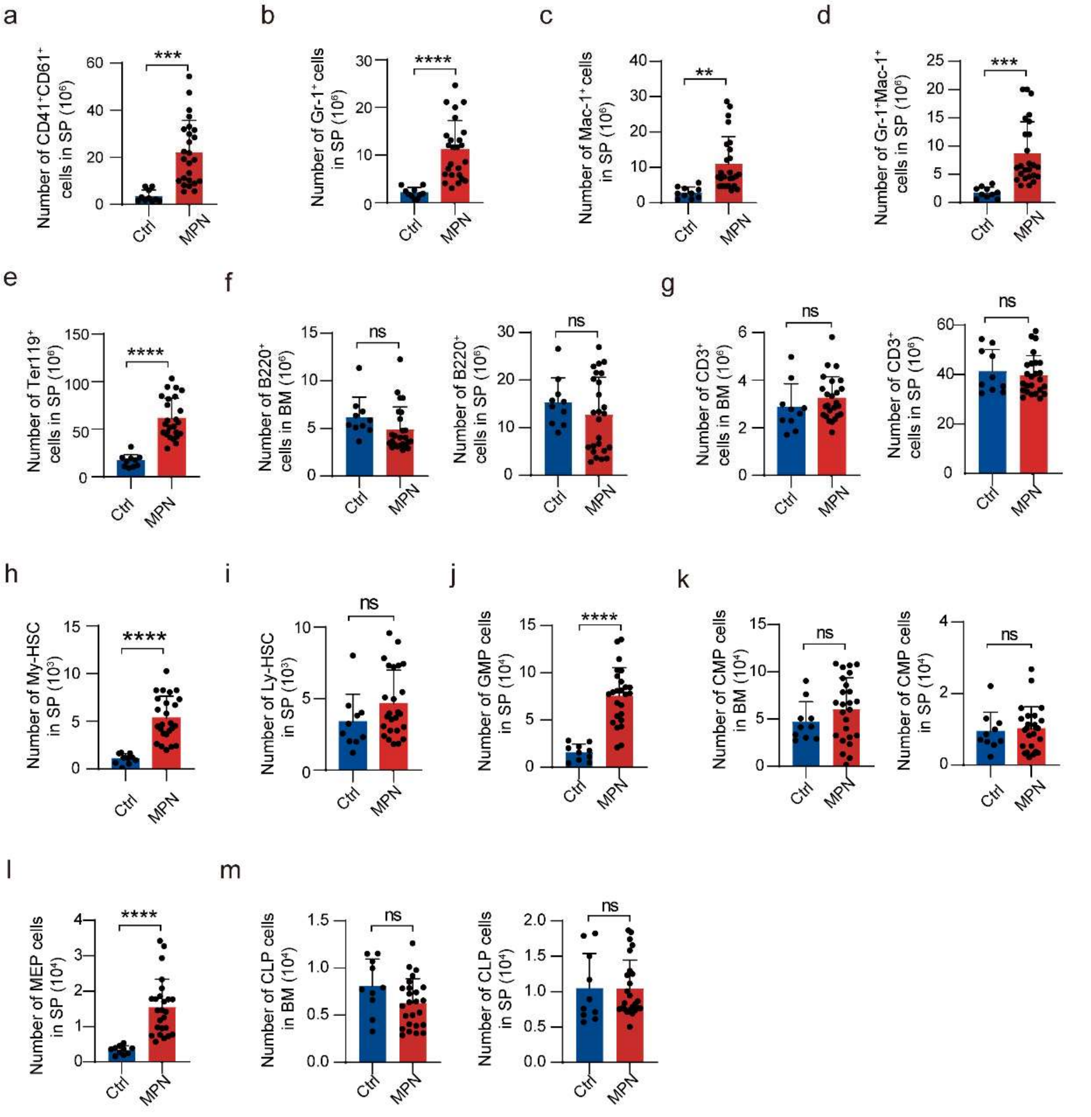

Supplementary Fig. 6 Hematological parameters and morphological characteristics of the BM and spleen from moribund mice with MPN.

(**a-e**) Absolute number of $CD41^{+}CD61^{+}$ (**a**), $Gr\text{-}1^{+}$(**b**), $Mac\text{-}1^{+}$(**c**), $Gr\text{-}1^{+}Mac\text{-}1^{+}$ (**d**), and $Ter119^{+}$ (**e**) cells in the spleen of moribund *Gadd45g* deficiency mice with MPN (n=25) and Ctrl mice (n=10).

(**f-g**) Absolute number of $B220^{+}$ (**f**) and $CD3^{+}$ (**g**) cells in the BM (left) and spleen (right) of moribund mice with MPN (n=25) and Ctrl mice (n=10).

(**h-j**) Absolute number of My-biased HSCs (**h**), Ly-biased HSCs (**i**) and GMP cells (**j**) in the spleen of moribund mice with MPN (n=25) and Ctrl mice (n=10).

(**k**) Absolute number of CMP cells in the BM (left) and spleen (right) of moribund mice with MPN (n=25) and Ctrl mice (n=10).

(**l**) Absolute number of MEP cells in the spleen of moribund mice with MPN (n=25) and Ctrl mice (n=10).

(**m**) Absolute number of CLP cells in the BM (left) and spleen (right) of moribund mice with MPN (n=25) and Ctrl mice (n=10).

Data are shown as means ± SD. **, $P < 0.01$; ***, $P < 0.001$; ****, $P < 0.0001$; ns, not significant (Two-tailed Student's *t* test).

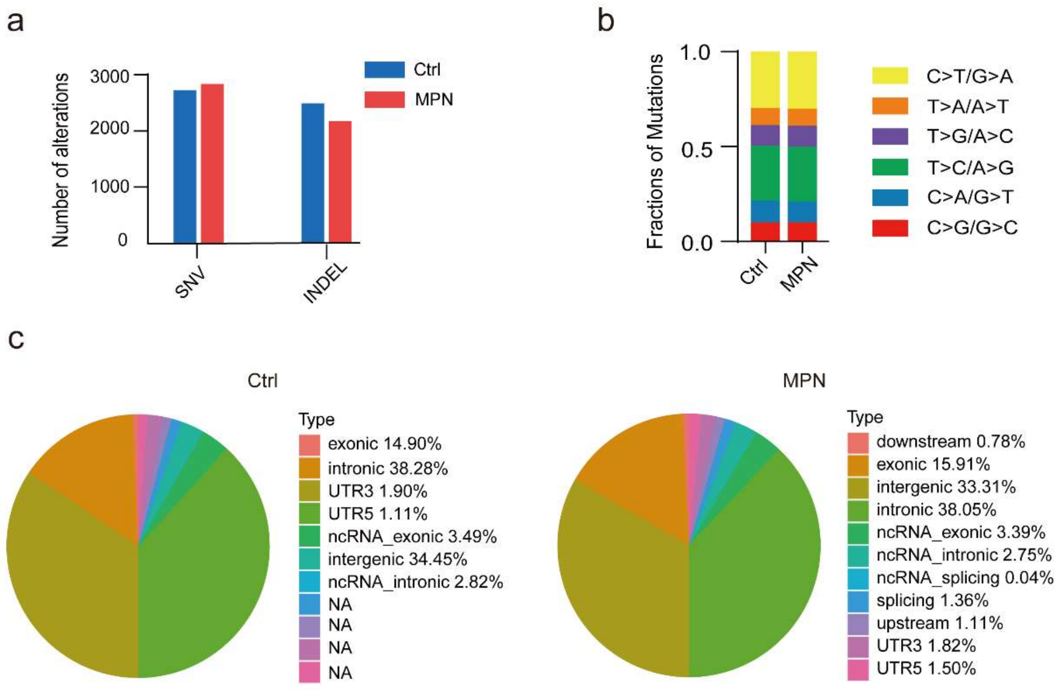

Supplementary Fig. 7 No malignancy-associated mutation is induced by *Gadd45g* deficiency.

(**a-c**) Whole-exome sequencing was performed on c-kit^{+} BM cells from 3 *Gadd45g*$^{+/-}$ mice with MPN, and those from their Ctrl. Mutational burden (**a**), mutation spectrum (**b**), and the percentages of different types of somatic substitutional mutations (**c**) were shown.

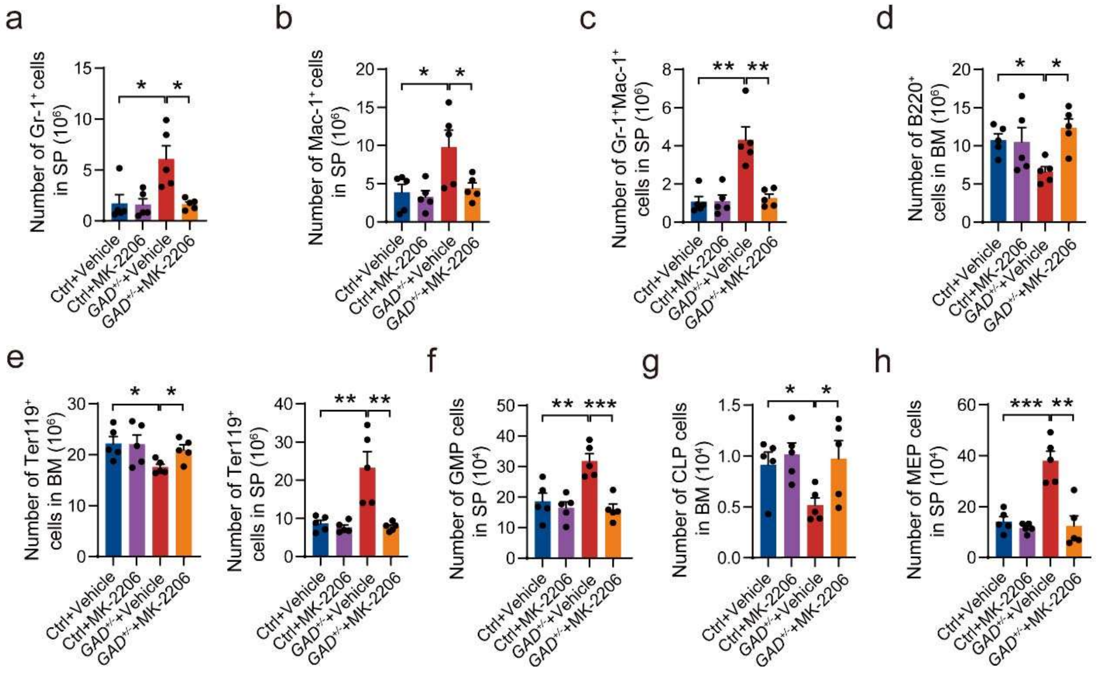

Supplementary Fig. 8 Inhibition of PI3K-AKT signaling pathway reverses the hematological abnormalities induced by *Gadd45g* deficiency in mice. (**a-h**) Two-month-old *Gadd45g*$^{+/-}$ and Ctrl mice were orally administered with vehicle or MK-2206 at 100mg/kg 3 times a week for 2 months, followed by once a week for another 2 months (No overt toxicity was observed at these doses and schedule). Absolute number of Gr-1^{+} (**a**), Mac-1^{+} (**b**), Gr-1^{+}Mac-1^{+} (**c**) cells in the spleen of mice in each group (n= 5 mice per group). Absolute number of B220^{+} in the BM of mice in each group (n= 5 mice per group) (**d**). Absolute number of Ter119^{+} cells in the BM (left) and spleen (right) of mice in each group (n= 5 mice per group) (**e**). Absolute number of GMP cells in the spleen of mice in each group (n= 5 mice per group) (**f**). Absolute number of CLP cells in the BM of mice in each group (n= 5 mice per group) (**g**). Absolute number of MEP

cells in the spleen of mice in each group (n= 5 mice per group) (**h**).

Data are shown as means ± SD. Multiple groups were analyzed with one-way ANOVA. *, $P < 0.05$; **, $P < 0.01$; ***, $P < 0.001$ (Two-tailed Student's *t* test).

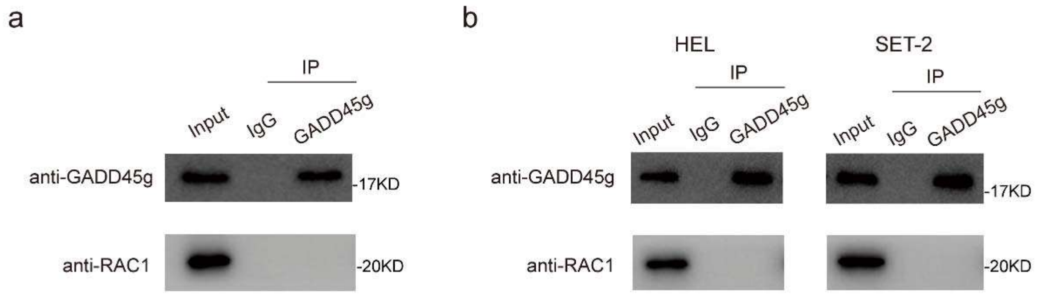

Supplementary Fig. 9 GADD45g shows no interaction with RAC1.

(**a**) c-kit$^+$ BM cells from wild type mice were lysed, precipitated with anti-GADD45g antibody, and detected by Western blot with anti-RAC1 and -GADD45g antibodies.

(**b**) HEL and SET-2 cells were lysed, precipitated with anti-GADD45g antibody, and detected by Western blot with anti-RAC1 and -GADD45g antibodies.

For **a-b**: Blots are representative of three independent experiments.

.

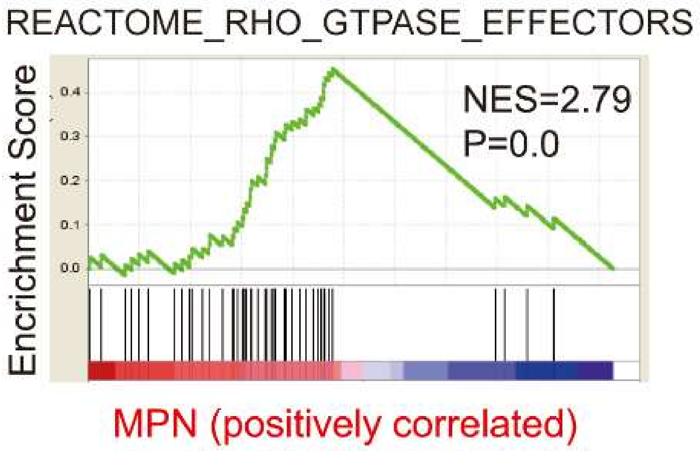

Supplementary Fig. 10 *Gadd45g* deficiency leads to activation of RHO-GTPase signaling pathway.

GSEA of our RNA-seq data showing positive enrichment of RHO-GTPase signaling pathway in *Gadd45g*-defficient cells from moribund mice with MPN. The normalized enrichment score (NES) and *P*-value was shown.

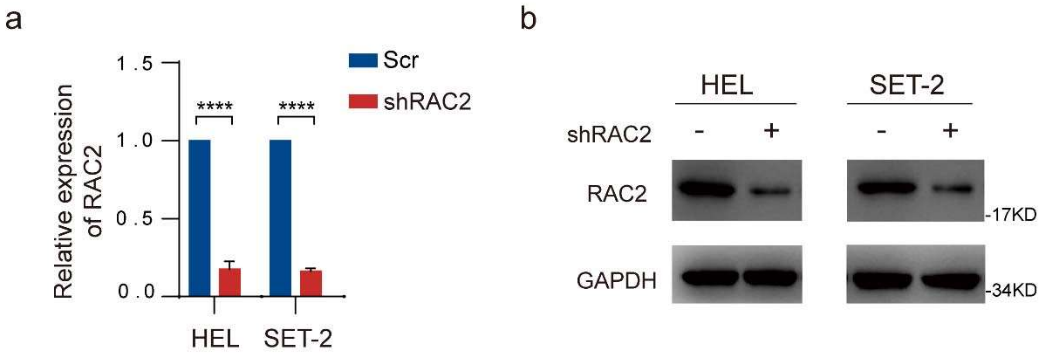

Supplementary Fig. 11 Knockdown efficiency of *RAC2* in the MPN cell lines.

(**a**) Relative expression of *RAC2* mRNA in HEL and SET-2 cells transfected with shRAC2 or scrambled control (Scr), as determined by qRT-PCR. Figures shown are representative of three independent experiments with similar results. Data are shown as mean ± SD (n=3 technical replicates). ****, $P < 0.0001$ (Two-tailed Student's *t* test).

(**b**) Western blot showing RAC2-protein levels in HEL and SET-2 cells after knockdown using shRAC2 compared to Scr. Blots are representative of three independent experiments.

Graphical representations of flow cytometry gating strategy for sorting LT-HSCs.

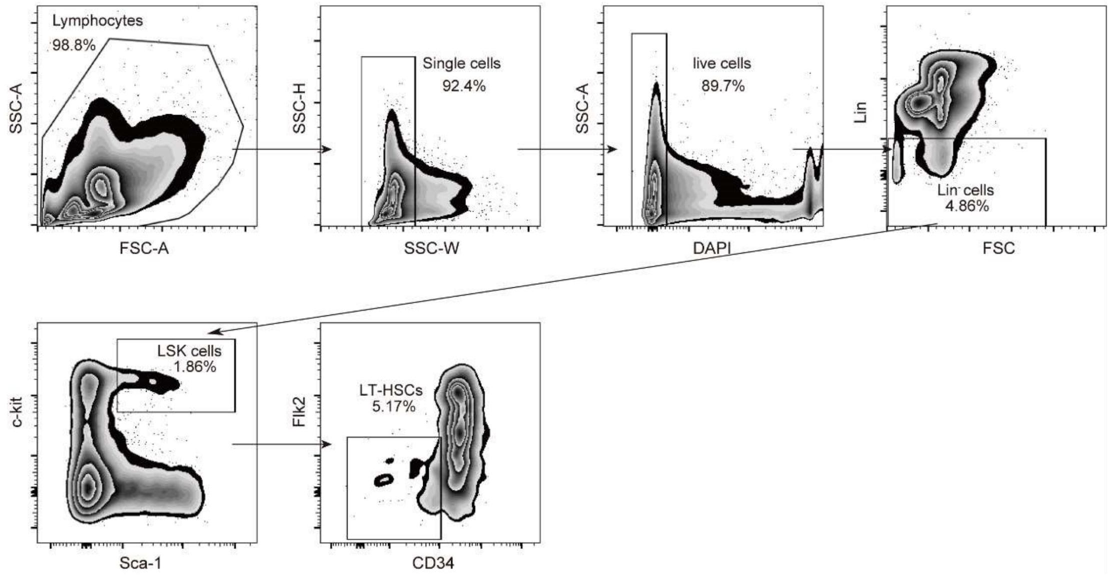

Graphical representations of flow cytometry sequential gating strategy for Fig. 2a, p-q, Fig. 4b (CD45.2$^+$ cells), Supplementary Fig. 4b.

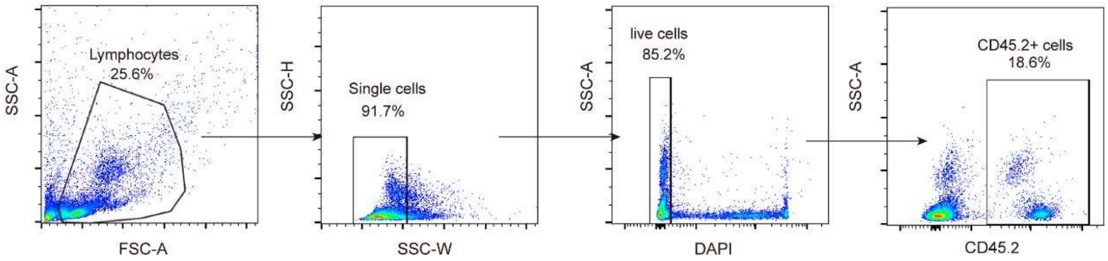

Graphical representations of flow cytometry sequential gating strategy for Fig. 2c, e, Fig. 3 i-l, Fig. 6d-f, Supplementary Fig. 5b, d, Supplementary Fig. 6b-e, Supplementary Fig. 8a-c, e.

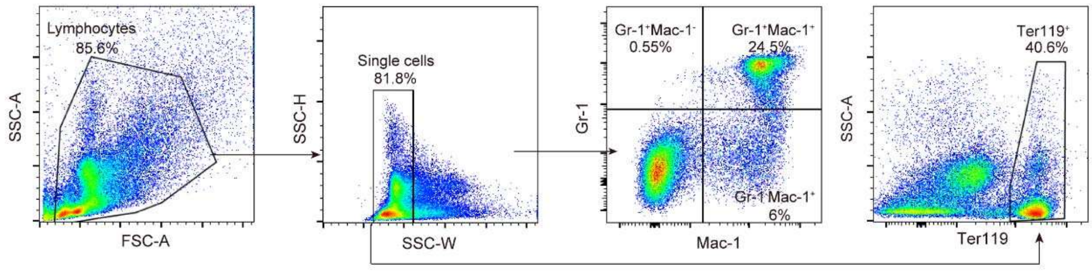

Supplementary Fig 12. **Graphical representations of flow cytometry gating strategies.**

Graphical representations of flow cytometry sequential gating strategy for Fig. 2d, Fig 4h-i, Supplementary Fig. 5c, Supplementary Fig. 6f-g, Supplementary Fig. 8d.

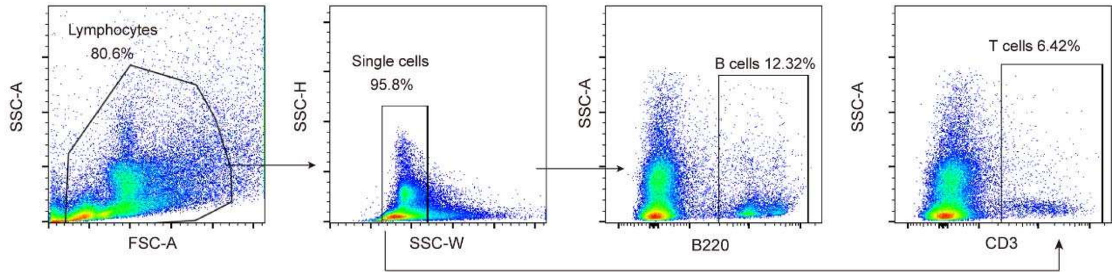

Graphical representations of flow cytometry sequential gating strategy for Fig. 2f, Fig. 3m-n, Fig. 6g, Supplementary Fig. 5e, Supplementary Fig. 6j-l, Supplementary Fig. 8f, h.

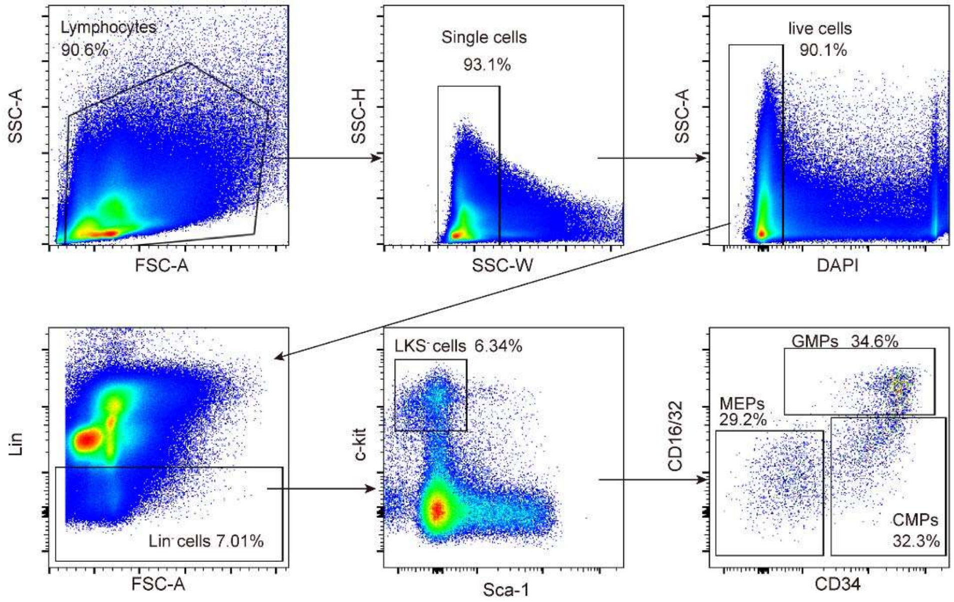

Supplementary Fig 12. **Graphical representations of flow cytometry gating strategies.**

Graphical representations of flow cytometry sequential gating strategy for Fig. 2g, Supplementary Fig. 5f, Supplementary Fig. 6m, Supplementary Fig. 8g.

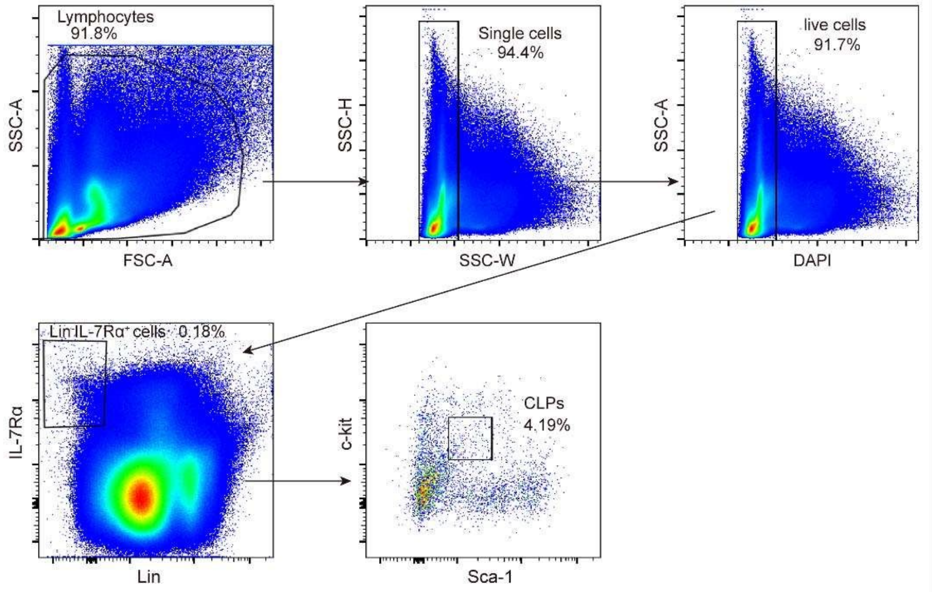

Graphical representations of flow cytometry sequential gating strategy for Fig. 2i-j.

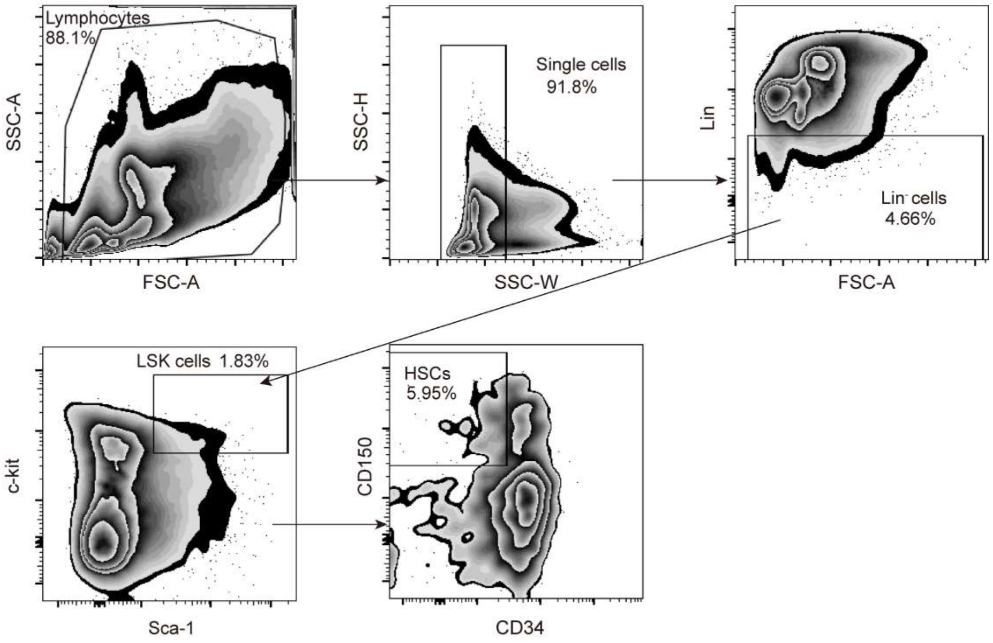

Supplementary Fig 12. Graphical representations of flow cytometry gating strategies.

Graphical representations of flow cytometry sequential gating strategy for Fig. 2l.

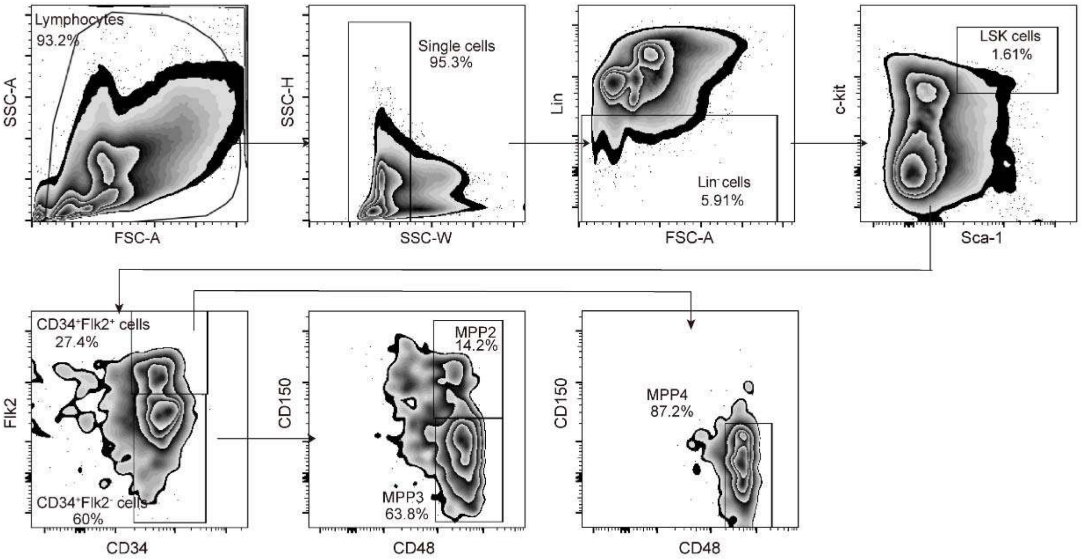

Graphical representations of flow cytometry sequential gating strategy for Fig. 2m.

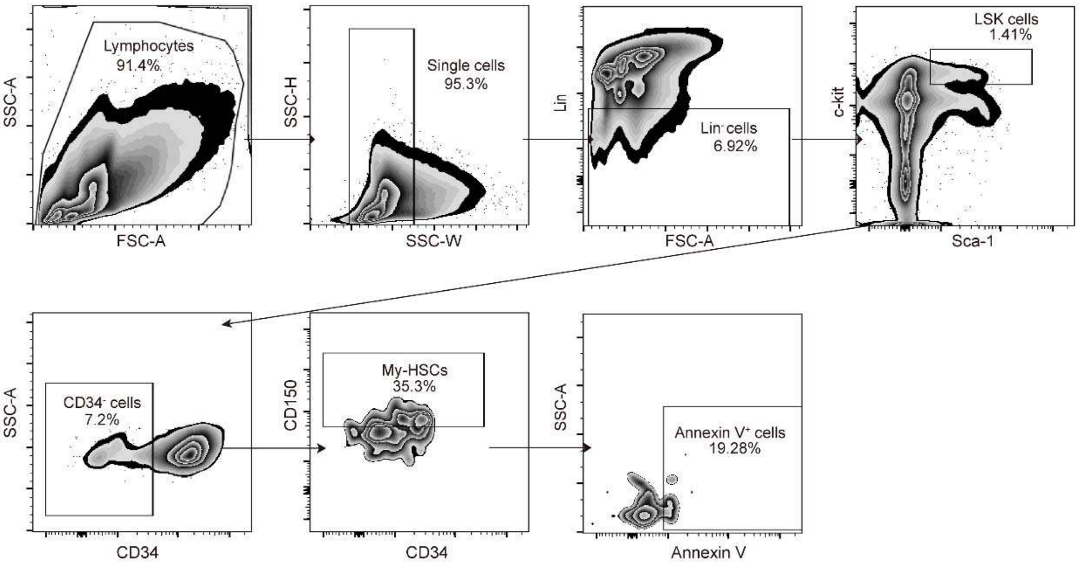

Supplementary Fig 12. **Graphical representations of flow cytometry gating strategies.**

Graphical representations of flow cytometry sequential gating strategy for Fig. 2n.

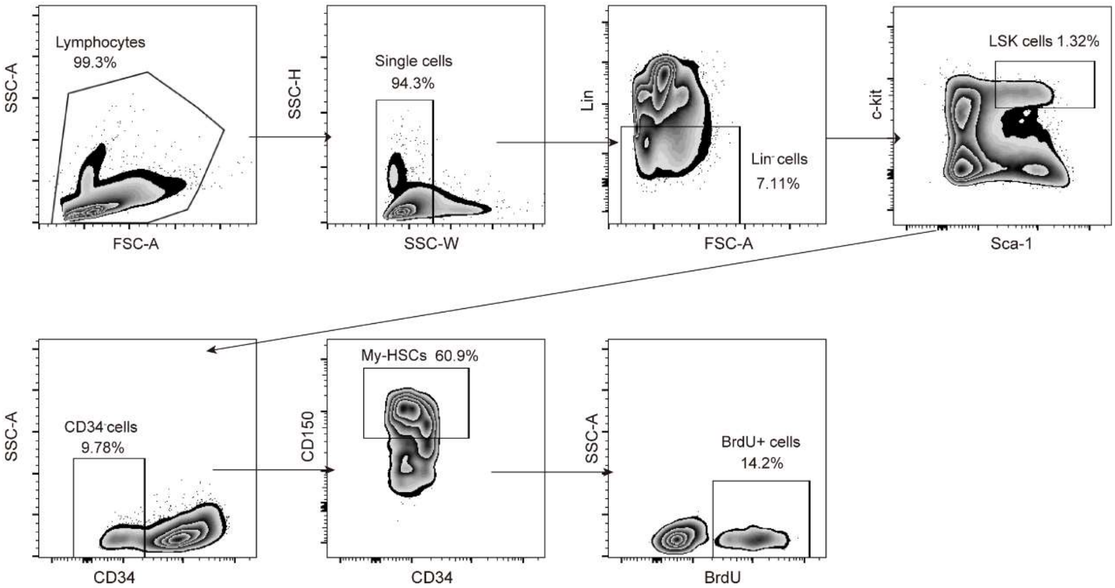

Graphical representations of flow cytometry sequential gating strategy for Fig. 2o.

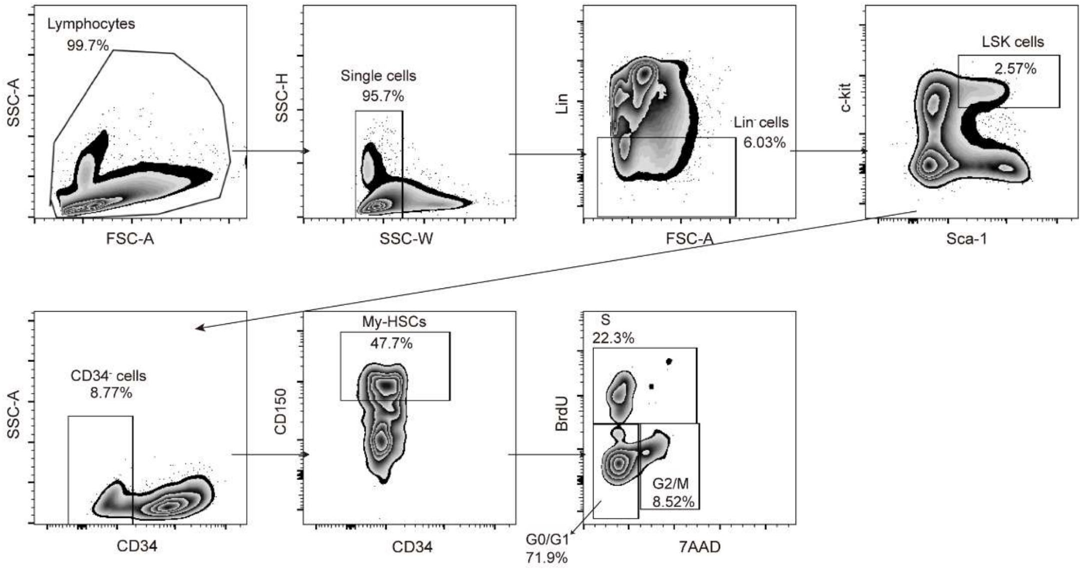

Supplementary Fig 12. **Graphical representations of flow cytometry gating strategies.**

Graphical representations of flow cytometry sequential gating strategy for Fig. 3h, Supplementary Fig. 6a.

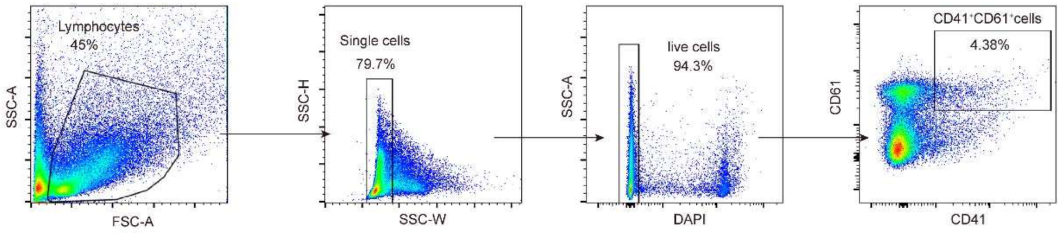

Graphical representations of flow cytometry sequential gating strategy for Fig 4b (myeloid, T, B cells), Supplementary Fig. 5g-h.

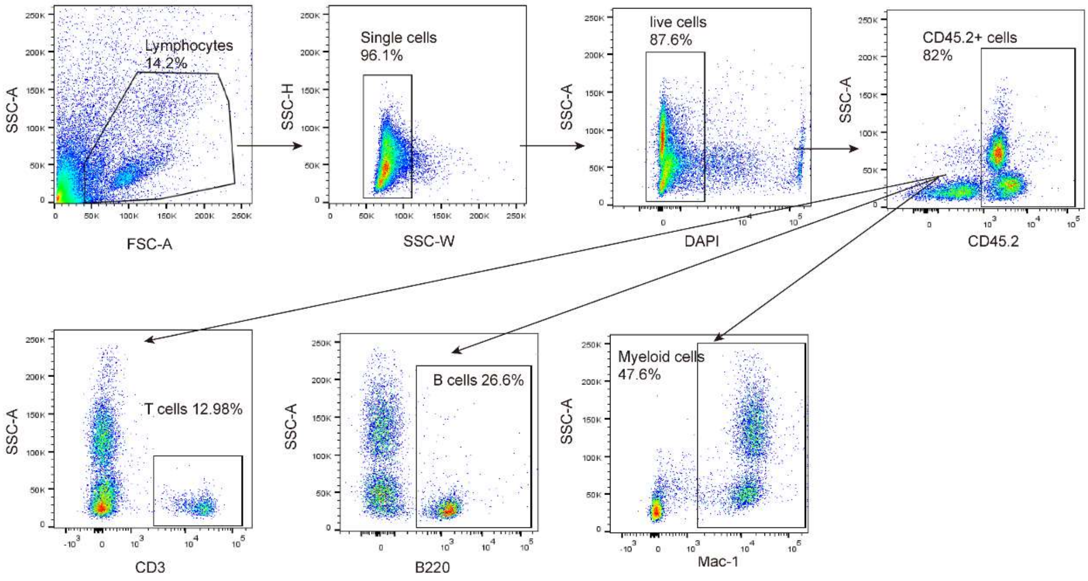

Supplementary Fig 12. **Graphical representations of flow cytometry gating strategies.**

Graphical representations of flow cytometry sequential gating strategy for Fig. 4e-g, j.

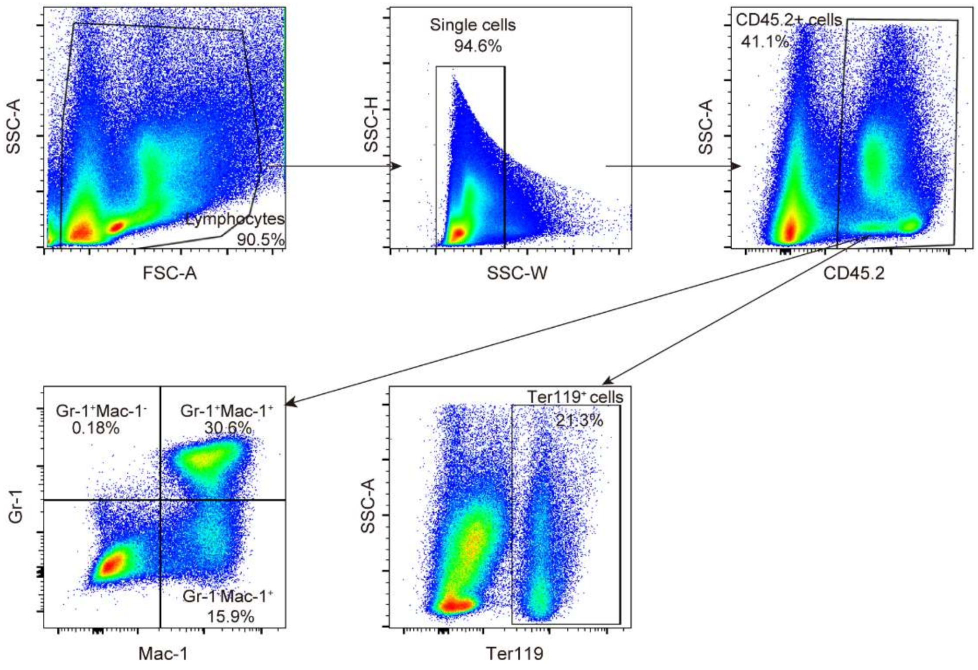

Graphical representations of flow cytometry sequential gating strategy for Fig. 4h-i.

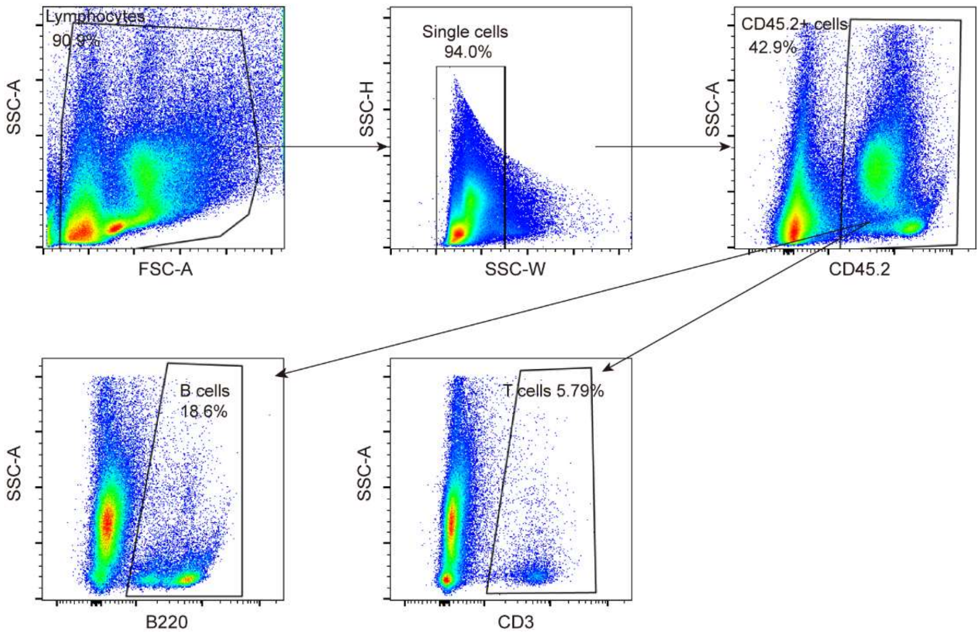

Supplementary Fig 12. **Graphical representations of flow cytometry gating strategies.**

Supplementary Table 1

Summary of tumor transfer experiments

**Donor cell**	**Morbidity (%)**	**Disease**	**Latency (days)**
**Noncompetitive transplantations**			
Ctrl	0	N/A	N/A
MPN	100%	MPN (5/5)	233-405
**Reintroduction of *Gadd45g***			
MPN Dox-	100%	MPN (6/6)	211-368
MPN Dox+	50%	MPN (3/6)	482-513

Supplementary Table 2

Combination of antibodies for flow cytometric analysis

Antibody	Color	Source	Clone number
CD45.2	PerCp-Cy5.5, PE	eBioscience (San Diego, CA, USA)	104
Ter119	PE/Cy7	eBioscience	TER-119
Gr-1	PE, APC/Cy7	eBioscience	1A8-Ly6g
Mac-1	APC, PE/Cy7	eBioscience	M1/70, ICRF44
CD3	PE	eBioscience	145-2C11
B220	PerCp/Cy5.5, APC	eBioscience	RA3-6B2
Lineage cocktail	Biotin	Miltenyi Biotec	53-7.3, M1/70.15.11.5, RA3-6B2, REA115, RB6-8C5, Ter-119
Streptavidin	APC/Cy7	BD Biosciences	N/A
c-Kit	BV605, APC, PerCP/Cy5.5, PE/Cy7	BD Biosciences	2B8
Sca-1	PE/Cy7, BV786	BD Biosciences	D7
CD34	FITC, eFlour 660, BV421	BD Biosciences	RAM34
CD135 (Flt-3)	PE, APC, BV421	BD Biosciences	A2F10.1
CD16/32 (FccRII/III)	PE, PerCP/Cy5.5	BD Biosciences	2.4G2
IL-7R	PE	BD Biosciences	SB/199
CD48	FITC, BV510	BD Biosciences	HM48-1
CD150	BV786, PE	BioLegend (San Diego, CA, USA)	TC15-12F12.2
hCD45	PE	BD Biosciences	HI30

Supplementary Table 3

Gating strategies for flow cytometric analysis

Target cells	Gating strategies
MPP2	$CD34^{+}Flk2^{-}$ $CD150^{+}$ $CD48^{+}$ Lin^{-} $c\text{-}Kit^{+}$ $Sca\text{-}1^{+}$
MPP3	$CD34^{+}Flk2^{-}$ $CD150^{-}$ $CD48^{+}$ Lin^{-} $c\text{-}Kit^{+}$ $Sca\text{-}1^{+}$
MPP4	$CD34^{+}Flk2^{+}$ $CD150^{-}$ $CD48^{+}$ Lin^{-} $c\text{-}Kit^{+}$ $Sca\text{-}1^{+}$
HSCs	$CD34^{-}CD150^{+}$ Lin^{-} $c\text{-}Kit^{+}$ $Sca\text{-}1^{+}$
LT-HSCs	$CD34^{-}Flk2^{-}$ Lin^{-} $c\text{-}Kit^{+}$ $Sca\text{-}1^{+}$
My-biased HSCs	$CD34^{-}CD150^{high}$ Lin^{-} $c\text{-}Kit^{+}$ $Sca\text{-}1^{+}$
Ly-biased HSCs	$CD34^{-}CD150^{low}$ Lin^{-} $c\text{-}Kit^{+}$ $Sca\text{-}1^{+}$
CMPs	$CD34^{+}$ $CD16/32^{-}$ Lin^{-} $c\text{-}Kit^{+}$ $Sca\text{-}1^{-}$
GMPs	$CD34^{+}$ $CD16/32^{+}$ Lin^{-} $c\text{-}Kit^{+}$ $Sca\text{-}1^{-}$
MEPs	$CD34^{-}$ $CD16/32^{-}$ Lin^{-} $c\text{-}Kit^{+}$ $Sca\text{-}1^{-}$
CLPs	Lin^{-} $IL\text{-}7R\alpha^{+}c\text{-}Kit^{low}Sca\text{-}1^{low}$
Granulocytes	$Gr\text{-}1^{+}$
Monocytes/macrophage	$Mac\text{-}1^{+}$
Neutrophils	$Gr\text{-}1^{+}Mac\text{-}1^{+}$
B lymphocytes	$B220^{+}$
T lymphocytes	$CD3^{+}$
Erythrocytes	$Ter119^{+}$
Megakaryocytes	$CD41^{+}CD61^{+}$

Supplementary Table 4

Oligonucleotide sequences used for constructs

Name	Forward	Reverse
pLKO.1-shGADD45g-1-GFP	CCGGCTCGACGCTGCAGATCCATTTCTCGAGAAATGGATCTGCAGCGCGATGTTTTTTG	AATTCAAAAACTCGACGCTGCAGATCCATTTCTCGAGAAATGGATCTGCAGCGCGATG
pLKO.1-shGADD45g-2-GFP	CCGGCCCGACAATGTGACCTTCTGTCTCGAGACAGAAGGTCACATTGTCGGGTTTTTG	AATTCAAAAACCCGACAATGTGACCTTCTGTCTCGAGACAGAAGGTCACATTGTCGGG
pLKO.1-shCtrl-GFP	CCGGGTCACCGTAGTCTCGTACACTCTCGAGAGTGTACGAGACTACGGTGACTTTTTTG	AATTCAAAAAGTCACCGTAGTCTCGTACACTCTCGAGAGTGTACGAGACTACGGTGAC
pLKO.1-shRAC2-Puro	CCGGAGGAGATTGACTCGGTGAAATCTCGAGATTTCACCGAGTCAATCTCCTTTTTTG	AATTCAAAAAAGGAGATTGACTCGGTGAAATCTCGAGATTTCACCGAGTCAATCTCCT
pLKO.1- shCtrl-Puro	CCGGGCTAAGATGGAGGACTAAGTTCTCGAGAACTTAGTCCTCCATCTTAGCTTTTTG	AATTCAAAAAGCTAAGATGGAGGACTAAGTTCTCGAGAACTTAGTCCTCCATCTTAGC
Plvx-tight-hGADD45g-GFP-Puro	TTGCGGCCGCAAGCCACCATGACTCTGGAAGAAGTCCGA	CCGGAATTCTCAGATTACAAGGATGACGACGATAAGCTCGGGGAGGGTGATGCT
Plvx-tight-mGadd45g- Puro	CCGGAATTCGCCACCATGACTCTGGAAGAAGTCCG	CCGCTCGAGTCACTCGGGAAGGGTGATGC
Tet-pLKO-puro-GADD45g	CCGGCCCGACAATGTGACCTTCTGTCTCGAGACAGAAGGTCACATTGTCGGGTTTTTG	AATTCAAAAACCCGACAATGTGACCTTCTGTCTCGAGACAGAAGGTCACATTGTCGGG

Supplementary Table 5

Sequences of forward and reverse primers used in the genotyping and qRT-PCR assays

Gene	Forward	Reverse
**Primers used for genotyping**		
Gadd45g	GGCCTTGTCTGGTAGTGGTCTTACA	CCTCATTCCCCATCACCCAGTCTCG
Vav-Cre	AGATGCCAGGACATCAGGAACCTG	ATCAGCCACACCAGACACAGAGATC
**Primers used for qRT-PCR**		
Human		
β-ACTIN	CGAGCGCGGCTACAGCTT	CCTTAATGTCACGCACGATT
GADD45g	GCCGGCGTCTACGAGTCA	CCAGCACACAGAAGGTCACATT
RAC2	CAACGCCTTTCCCGGAGAG	TCCGTCTGTGGATAGGAGAGC
Mouse		
Gadd45g	TTCGTGGTCGACACAATGACT	GGACTTTGGCGGACTCGTAGA
Gadd45a	CCGAAAGGATGGACACGGTG	TTATCGGGGTCTACGTTGAGC
Gadd45b	CAACGCGGTTCAGAAGATGC	GGTCCACATTCATCAGTTTGGC

Supplementary Table 6

Primers used for Methylation-specific PCR and ChIP-qPCR

Gene	Forward	Reverse
	MS-PCR	
GADD45g (Methylation)	TATTTAGTTTCGATTTTACGCGT	AAAACGAACAAATTACGACCG
GADD45g (Unmethylation)	TATTTAGTTTTGATTTTATGTGT	AAAACAAACAAATTACAACCA
	ChIP-PCR	
GADD45g (+567-+6)	GCCAGGCGAGATGAAATCTG	TGATGTAAATGAGGCGGCGAG
