# Supplementary material for: Gadd45g insufficiency drives the pathogenesis of myeloproliferative neoplasms
Source: Nat Commun. 2024 Apr 6;15:2989. doi: 10.1038/s41467-024-47297-2 (PMC10998908; doi:10.1038/s41467-024-47297-2)
Supplement: Supplementary file 3 — Description of Additional Supplementary Files [file 41467_2024_47297_MOESM3_ESM.pdf]

Description of Additional Supplementary Files

File Name: Supplementary Data 1

Description: Proteins detected by LC-MS/MS by anti-GADD45g IP in c-kit+ BM cells of wild-type mice.
