# Supplementary material for: Gadd45g insufficiency drives the pathogenesis of myeloproliferative neoplasms
Source: Nat Commun. 2024 Apr 6;15:2989. doi: 10.1038/s41467-024-47297-2 (PMC10998908; doi:10.1038/s41467-024-47297-2)
Supplement: Supplementary file 5 — Reporting Summary [file 41467_2024_47297_MOESM5_ESM.pdf]

nature portfolio

Corresponding author(s): Xiaotong Ma, Tao Cheng, Zhijian Xiao, Lei Zhang

Last updated by author(s): Mar 8, 2024

Reporting Summary

Nature Portfolio wishes to improve the reproducibility of the work that we publish. This form provides structure for consistency and transparency in reporting. For further information on Nature Portfolio policies, see our Editorial Policies and the Editorial Policy Checklist.

Statistics

For all statistical analyses, confirm that the following items are present in the figure legend, table legend, main text, or Methods section.

n/a	Confirmed	
☐	☒	The exact sample size (*n*) for each experimental group/condition, given as a discrete number and unit of measurement
☐	☒	A statement on whether measurements were taken from distinct samples or whether the same sample was measured repeatedly
☐	☒	The statistical test(s) used AND whether they are one- or two-sided<br>*Only common tests should be described solely by name; describe more complex techniques in the Methods section.*
☒	☐	A description of all covariates tested
☐	☒	A description of any assumptions or corrections, such as tests of normality and adjustment for multiple comparisons
☐	☒	A full description of the statistical parameters including central tendency (e.g. means) or other basic estimates (e.g. regression coefficient) AND variation (e.g. standard deviation) or associated estimates of uncertainty (e.g. confidence intervals)
☐	☒	For null hypothesis testing, the test statistic (e.g. *F*, *t*, *r*) with confidence intervals, effect sizes, degrees of freedom and *P* value noted<br>*Give P values as exact values whenever suitable.*
☒	☐	For Bayesian analysis, information on the choice of priors and Markov chain Monte Carlo settings
☒	☐	For hierarchical and complex designs, identification of the appropriate level for tests and full reporting of outcomes
☒	☐	Estimates of effect sizes (e.g. Cohen's *d*, Pearson's *r*), indicating how they were calculated

Our web collection on statistics for biologists contains articles on many of the points above.

Software and code

Policy information about availability of computer code

Data collection	SYSMEX XN-1000 Sysmex's flagship analyzer for blood cell count;<br>FACS Canto™II or FACS LSRII or FACS AriaIII (BD) for FACS;<br>chemiDoc (Biorad) for Western blot;<br>ABI 7500 Sequence Detection System for qRT-PCR;<br>UltraVIEW VoX spinning disk confocal system (Perkinelmer) for immunofluorescence;<br>Bio-Plex MAGPIX (Luminex/Bio-Rad) for cytokine quantification;<br>The RNA sequencing library was prepared and sequenced on Illumina HiSeq 4000 platform;<br>Whole exome sequencing data was obtained using Illumina Novaseq 6000 platform;<br>Mice were imaged utilizing Caliper IVIS Lumina II in vivo imaging system (Caliper Life Sciences, Hopkinton, Massachusetts, USA).
Data analysis	FACSDiva software v 6.1.3 (BD) or FlowJo software v10.6.2 (Tree Star) for analysis of FACS results;<br>Figures with statistic were generated with GraphPad Prism v8;<br>Image J v1.42;<br>QuantStudioTM Design & Analysis Software for gene expression analysis.

For manuscripts utilizing custom algorithms or software that are central to the research but not yet described in published literature, software must be made available to editors and reviewers. We strongly encourage code deposition in a community repository (e.g. GitHub). See the Nature Portfolio guidelines for submitting code & software for further information.

Data

Policy information about availability of data

All manuscripts must include a data availability statement. This statement should provide the following information, where applicable:

- Accession codes, unique identifiers, or web links for publicly available datasets
- A description of any restrictions on data availability
- For clinical datasets or third party data, please ensure that the statement adheres to our policy

The main data supporting the findings of the study are available within the manuscript and Supplementary Information files. RNA-Seq data have been deposited in NCBI's GEO repository under accession number GSE229495 [https://www.ncbi.nlm.nih.gov/geo/query/acc.cgi?acc=GSE229495]. WES data have been deposited in NCBI's Sequence Read Archive under accession number PRJNA918529 [https://www.ncbi.nlm.nih.gov/bioproject/PRJNA918529]. The mass spectrometry proteomics data have been deposited to the ProteomeXchange Consortium via the iProX partner repository with the dataset identifier PXD039261 [https://www.iprox.cn//page/project.html?id=IPX0005710000].

Research involving human participants, their data, or biological material

Policy information about studies with human participants or human data. See also policy information about sex, gender (identity/presentation), and sexual orientation and race, ethnicity and racism.

Reporting on sex and gender	Bone marrow specimens from healthy volunteers and patients with newly diagnosed MPNs: female and male ; Human cord blood: healthy postpartum women.
Reporting on race, ethnicity, or other socially relevant groupings	We do not use or collect data about ethnicity.
Population characteristics	Bone marrow specimens were collected from healthy adult donors and patients with newly diagnosed MPNs at the Institute of Hematology and Blood Diseases Hospital, Chinese Academy of Medical Sciences. The patient cohort consisted of 21 males and 14 females with a median age of 53 years (range: 14-72 years). 16 patients were diagnosed with ET, 19 patients were diagnosed with PV. Human cord blood (CB) was obtained from healthy postpartum women. Sex and/or gender of participants was determined based on self-report.
Recruitment	Healthy adult donors and patients with newly diagnosed MPNs at the Institute of Hematology and Blood Diseases Hospital were recruited. To protect donors' privacy, samples have been de-identified. There are no known biases affecting patient recruitment, selection of samples, or in analysis.
Ethics oversight	All primary samples from human subjects were obtained after informed consent. All laboratory experiments with primary samples were conducted according to the ethical principles for medical research and approved by the Ethics Committee of the Institute of Hematology and Blood Diseases Hospital.

Note that full information on the approval of the study protocol must also be provided in the manuscript.

Field-specific reporting

Please select the one below that is the best fit for your research. If you are not sure, read the appropriate sections before making your selection.

☒ Life sciences ☐ Behavioural & social sciences ☐ Ecological, evolutionary & environmental sciences

For a reference copy of the document with all sections, see nature.com/documents/nr-reporting-summary-flat.pdf

Life sciences study design

All studies must disclose on these points even when the disclosure is negative.

Sample size	For in vivo experiments, sample sizes were determined based on our previous experience (Guo D, et al. Blood, 2021 Aug 12;138(6):464-479, doi: 10.1182/blood.2020008229; Nan W, et al. Blood, 2021 May 27;137(21):2907-2919, doi: 10.1182/blood.2020007489), which was sufficient to generate statistically significant results (p value 0.05). For in vitro experiments, at least three independent biological replicates were performed. Such sample sizes are typical for the in vitro experiments and sufficient for a statistical analysis. Sizes of human samples were based on the sample availability and the consistency of measurable differences between groups. No statistical method was used to predetermine sample size.
Data exclusions	All data were included in this study.
Replication	Each experiment in this study was independently repeated for at least 3 times to generate final conclusion. All attempts at replication were successful yielding similar results.
Randomization	For in vivo experiments, mice were randomly allocated to each group. For cell line-based experiments, randomization was not required because all samples were analyzed equally.

Blinding

For most of the animal experiments, investigators were not blinded to group allocation as they needed to be aware of the mouse genotypes during treatment steps. Blinding was implemented during Wright-Giemsa and H&E staining, as well as in the quantification of bioluminescence imaging. In human experiments, blinding was not feasible during sample collection, as investigators needed to document the types of diseases. Researchers were blinded to the expression levels of GADD45g when performing the colony formation assay. Blinding was impractical for cell experiments since the investigators responsible for group allocation were also involved in performing treatments, sample processing, data collection, and data analysis

Reporting for specific materials, systems and methods

We require information from authors about some types of materials, experimental systems and methods used in many studies. Here, indicate whether each material, system or method listed is relevant to your study. If you are not sure if a list item applies to your research, read the appropriate section before selecting a response.

Materials & experimental systems

n/a	Involved in the study
☐	☒ Antibodies
☐	☒ Eukaryotic cell lines
☒	☐ Palaeontology and archaeology
☐	☒ Animals and other organisms
☒	☐ Clinical data
☒	☐ Dual use research of concern
☒	☐ Plants

Methods

n/a	Involved in the study
☒	☐ ChIP-seq
☐	☒ Flow cytometry
☒	☐ MRI-based neuroimaging

Antibodies

Antibodies used

Antibodys for FACS:
CD45.2-PerCp-Cy5.5 (45045482, eBioscience, clone 104)
CD45.2-PE (56045481, eBioscience, clone 104)
Ter119-PE-Cy7 (25592182, eBioscience, clone TER-119)
Gr-1-PE (12966882, eBioscience,clone 1A8-Ly6g)
Mac-1-APC (47011282, eBioscience, clone M1/70)
Mac-1-PE-Cy7 (25011842, eBioscience, clone ICRF44)
CD3-PE (12003182, eBioscience, clone 145-2C11)
B220-PerCp-Cy5.5 (45045282, eBioscience, clone RA3-6B2)
B220-APC (47045282, eBioscience, clone RA3-6B2)
Lineage cocktail Biotin (130092613, Miltenyi Biotec, clone 53-7.3, M1/70.15.11.5, RA3-6B2, REA115, RB6-8C5, Ter-119)
Streptavidin-APC-Cy7 (554063, BD Biosciences)
c-Kit-BV605 (563146, BD Biosciences, clone 2B8)
c-Kit-APC (553356, BD Biosciences, clone 2B8)
c-Kit-PerCp-Cy5.5 (560557, BD Biosciences, clone 2B8)
c-Kit-PE-Cy7 (561681, BD Biosciences, clone 2B8)
Sca-1-PE-Cy7 (561021, BD Biosciences, clone D7)
Sca-1-BV786 (563991, BD Biosciences, clone D7)
CD34-FITC (560238, BD Biosciences, clone RAM34)
CD34-BV421 (562608, BD Biosciences, clone RAM34)
CD135 (Flt-3)-PE (553842, BD Biosciences, clone A2F10.1)
CD135 (Flt-3)-APC (560718, BD Biosciences, clone A2F10.1)
CD135 (Flt-3)-BV421 (562898, BD Biosciences, clone A2F10.1)
CD16/32-PE (553145, BD Biosciences, clone 2.4G2)
CD16/32-PerCp-Cy5.5 (560540, BD Biosciences, clone 2.4G2)
IL-7R-PE (552543, BD Biosciences, clone SB/199)
CD48-FITC (557484, BD Biosciences, clone HM48-1)
CD48-BV510 (563536, BD Biosciences, clone HM48-1)
CD150-BV785 (115937, BioLegend, clone TC15-12F12.2)
CD150-PE (162605, BioLegend, clone TC15-12F12.2)
hCD45-PE (555483, BD Biosciences, clone HI30)

Antibodys for Western blot:
GADD45g (sc393261, Santa Cruz, clone B-1)
AKT (9272S, Cell Signaling Technology, Polyclonal)
AKT phospho-Ser473 (4060, Cell Signaling Technology, clone D9E)
PI3K (ab154598, Abcam, polyclonal)
PI3K phosphor-Y607 (ab182651, Abcam, polyclonal)
PAK1 (2602, Cell Signaling Technology, Polyclonal)
PAK1 phospho-Ser144 (2606, Cell Signaling Technology, Polyclonal)
RAC2 (ab191527, Abcam, polyclonal)
GAPDH (2118, Cell Signaling Technology, clone 14C10)
JAK2 (3230, Cell Signaling Technology, clone D2E12)

phospho-JAK2 (Tyr1007/1008) (3776, Cell Signaling Technology, clone C80C3)
β-Actin (sc-81178, Santa Cruz, clone ACTBD11B7)

Antibodys for immunofluorescence:
GADD45g (sc393261, Santa Cruz, clone B-1)
RAC2 (abx001053, Abbexa, Polyclonal)
RAC1 (ab97732, Abcam, polyclonal)

Antibody for chip assay:
Anti-acetyl-Histone H3 (Lys9) (9649, Cell Signaling Technology, clone C5B11)
Anti-acetyl-Histone H4 (acetyl K8) (ab45166, Abcam, clone EP1002Y)
Normal rabbit IgG antibodies (ab172730, Abcam clone EPR25A)

Validation

The antibodies used in this work were purchased from companies, and validated by the manufacturers and by extensive use in published work.
The antibodies used for flow cytometry in this manuscript are commonly used, commercially available clones. The primary antibody vendors used in this project (eBioscience, BD Biosciences, Biolegend, and Miltenyi Biotec) carry out extensive validation processes for flow cytometry antibodies. These are selected from high-affinity clones, tested for binding across multiple assays.
Other antibodies used for western blot, immunofluorescence, Immunoprecipitation and chip assay have been validated by the manufacturer (Cell Signaling Technology, Santa-Cruz, Abcam and Abbexa), Validation certificates can be found on the manufacturer's websites as follows:
Anti-AKT (9272S):https://www.cellsignal.cn/products/primary-antibodies/akt-antibody/9272
Anti-AKT phospho-Ser473(4060): https://www.cellsignal.cn/products/primary-antibodies/phospho-akt-ser473-d9e-xp-174-rabbit-mab/4060
Anti-PAK1 (2602):https://www.cellsignal.cn/products/primary-antibodies/pak1-antibody/2602
Anti-PAK1 phospho-Ser144 (2606):https://www.cellsignal.cn/products/primary-antibodies/phospho-pak1-ser144-pak2-ser141-antibody/2606
Anti-GAPDH (2118):https://www.cellsignal.cn/products/primary-antibodies/gapdh-14c10-rabbit-mab/2118
Anti-JAK2 (3230):https://www.cellsignal.cn/products/primary-antibodies/jak2-d2e12-xp-174-rabbit-mab/3230
Anti-phospho-JAK2 (Tyr1007/1008) (3776):https://www.cellsignal.cn/products/primary-antibodies/phospho-jak2-tyr1007-1008-c80c3-rabbit-mab/3776
Anti-acetyl-Histone H3 (Lys9) (9649):https://www.cellsignal.cn/products/primary-antibodies/acetyl-histone-h3-lys9-c5b11-rabbit-mab/9649
Anti-GADD45g (sc393261):https://www.scbt.com/p/gadd-45gamma-antibody-b-1?requestFrom=search
Anti-β-Actin (sc-81178):https://www.scbt.com/p/beta-actin-antibody-actbd11b7?requestFrom=search
Anti-PI3K (ab154598):https://www.abcam.cn/products/primary-antibodies/pi-3-kinase-catalytic-subunit-gammapi3k-gamma-antibody-ab154598.html
Anti-PI3K phosphor-Y607 (ab182651):https://www.abcam.cn/products/primary-antibodies/pi-3-kinase-p85-alpha-phospho-y607-antibody-ab182651.html
Anti-RAC2 (ab191527):https://www.abcam.cn/products/primary-antibodies/rac2-antibody-ab191527.html
Anti-RAC1 (ab97732):https://www.abcam.cn/products/primary-antibodies/rac1-antibody-ab97732.html
Anti-acetyl-Histone H4 (acetyl K8) (ab45166):https://www.abcam.cn/products/primary-antibodies/histone-h4-acetyl-k8-antibody-ep1002y-chip-grade-ab45166.html
Normal rabbit IgG antibodies (ab172730):https://www.abcam.cn/products/primary-antibodies/rabbit-igg-monoclonal-epr25a-isotype-control-ab172730.html
Anti-RAC2 (abx001053):https://www.abbexa.com/rac2-antibody-p-39939

Eukaryotic cell lines

Policy information about cell lines and Sex and Gender in Research

Cell line source(s)

HEL (TIB-180) and SET-2 (ACC 608) cells were obtained from American Type Culture Collection (ATCC) and German Collection of Microorganisms and Cell Cultures, respectively. Luciferase-expressing HEL92.1.7 cells (HEL92.1.7-Luc cells, NM-B24-TG01) were purchased from Shanghai Model Organisms Center, Inc. (Shanghai, China). 293T cells (CRL-11268) were obtained from ATCC.

Authentication

Authentication and associated testing were performed by the vendors.
HEL, SET-2 and 293T cell lines were validated using STR analysis by the vendors. Luciferase activity assay was performed before inoculating HEL92.1.7-Luc cells by the vendors.

Mycoplasma contamination

HEL, SET-2 ,293T and HEL92.1.7-Luc cell lines were tested negative for mycoplasma contamination.

Commonly misidentified lines
(See ICLAC register)

No commonly misidentified line was involved in this study.

Animals and other research organisms

Policy information about studies involving animals; ARRIVE guidelines recommended for reporting animal research, and Sex and Gender in Research

Laboratory animals

Gadd45gflox/WTmice (6-8 weeks old, 5 females, C57BL/6N strain) were purchased from Biocytogen Pharmaceuticals (Beijing, China). C57BL/6J and C57B6.SJL mice (6-8 weeks old, male and female) were purchased from the animal facility of the State Key Laboratory of Experimental Hematology (SKLEH). NOD-PrkdcscidIL2rgtm1(NSG) mice (6-8 weeks old, 40 females, strain# N-000002) were purchased from Beijing HFK Bio-Technology Co. Ltd (Beijing, China). B6.Cg-Commd10Tg(Vav1-icre)A2Kio/J (Vav-Cre) mice (6-8 weeks

	old, 2 males and 2 females, strain# 008610) were purchased from Jakson Lab. Gadd45gflox/WT mice were crossed with Vav-Cre mice to generate Gadd45gflox/WT; Vav-Cre and Gadd45gflox/flox; Vav-Cre mice. Mice were maintained at macroenvironmental temperature of 21-22°C, humidity (48-52%), in a conventional 12:12 light/dark cycle with lights on at 6:00 a.m. and off at 6:00 p.m. All animal studies were approved by the Institutional Animal Care and Use Committees of SKLEH. All mice were kept under specific pathogen-free conditions with free access to food and water in accordance to Swiss federal regulations.
Wild animals	No wild animals were used in this study.
Reporting on sex	The findings apply to both sexes.
Field-collected samples	This study did not involve samples collected from field.
Ethics oversight	All animal experiments were performed in compliance with institutional guidelines and approved by the Institutional Animal Care and Use Committees of SKLEH.

Note that full information on the approval of the study protocol must also be provided in the manuscript.

Flow Cytometry

Plots

Confirm that:

☒ The axis labels state the marker and fluorochrome used (e.g. CD4-FITC).

☒ The axis scales are clearly visible. Include numbers along axes only for bottom left plot of group (a 'group' is an analysis of identical markers).

☒ All plots are contour plots with outliers or pseudocolor plots.

☒ A numerical value for number of cells or percentage (with statistics) is provided.

Methodology

Sample preparation	The bone marrow cell suspensions were flushed from femurs and tibiae. Spleen cells were pestled by the plug of a 10 mL syringe and then filtered. Cells were centrifuged and resuspended in incubation buffer (PBS containing 2% FBS) with primary antibodies for 30 minutes on ice. Cells were then washed with incubation buffer and incubated for 40 minutes with secondary antibodies on ice. After washing, cells were resuspended in incubation buffer, filtered through mesh and analyzed.
Instrument	FACS Canto™II, FACS LSRII, FACS AriaIII
Software	FlowJo software v10.6.2 (Tree Star)
Cell population abundance	The frequencies of the key cell populations are shown in the figures. Fig. 1c shows the gates and frequencies of myeloid-biased (My-biased) and lymphoid-biased (Ly-biased) hematopoietic stem cells. Supplementary Fig. 4f shows the gates and frequencies of granulocyte/monocyte progenitors (GMP), common myeloid progenitors (CMP) and megakaryocyte/erythroid progenitors (MEP). Fig. 4j and Supplementary Fig. 8d show the gates and frequencies of apoptotic cells. Fig. 4k shows the gates and frequencies of cells in different phases of cell cycle.
Gating strategy	Cell debris was excluded using FSC/SSC gates, aggregates were excluded using SSC-H/SSC-W gates. Boundaries between positive and negative staining cell populations were defined by using fluorescence minus one controls.

☒ Tick this box to confirm that a figure exemplifying the gating strategy is provided in the Supplementary Information.
